# Coronary CT angiography derived FFR in patients with left main disease

**DOI:** 10.1007/s10554-021-02371-4

**Published:** 2021-08-12

**Authors:** Katharina A. Riedl, Jesper M. Jensen, Brian S. Ko, Jonathon Leipsic, Erik L. Grove, Ole N. Mathiassen, Hans Erik Bøtker, Bjarne L. Nørgaard

**Affiliations:** 1 Department of Cardiology, University Heart & Vascular Center Hamburg, Martinistraße 52, 20246 Hamburg, Germany; 2 Department of Cardiology, Aarhus University Hospital – Skejby, Aarhus, Palle Juul-Jensens Boulevard 69, 8200 Aarhus N, Denmark; 3 Monash Cardiovascular Research Centre, Monash University and Monash Heart, Monash Health, 246 Clayton Rd, Clayton, VIC 3168 Australia; 4 Department of Radiology, St. Paul’s Hospital, University of British Columbia, 1081 Burrard St., Vancouver, BC V6Z1Y6 Canada

**Keywords:** Computed tomography angiography, Coronary angiography, Coronary artery disease, Fractional flow reserve, Left main

## Abstract

The presence of left main coronary artery disease (LMCAD) is associated with an unfavorable clinical outcome. The clinical utility of FFR_CT_ testing for non-invasive physiological assessment in LMCAD remains largely unknown. In this single center observational study LMCAD patients were retrospectively identified between November 2015 and December 2017. We evaluated the relationship between LMCAD diameter stenosis and downstream FFR_CT_ values, and the clinical consequences following FFR_CT_ testing in patients with LMCAD. The composite endpoint (all-cause death, myocardial infarction, unplanned revascularization) was determined over a median follow-up of 1.1 years. LMCAD was registered in 432 of 3202 (13%) patients having coronary CTA. FFR_CT_ was prescribed in 213 (49%), while 59 (14%) patients were referred directly to invasive angiography or myocardial perfusion imaging. FFR_CT_ was performed in 195 (45%) patients. LM stenosis severity was inversely related to downstream FFR_CT_ values. In patients with simple LMCAD with stenosis ≥ 50%, > 80% had FFR_CT_ > 0.80 in non-diseased proximal and downstream segments (n = 7). No patients with simple LMCAD and FFR_CT_ > 0.80 (n = 20) suffered an adverse clinical outcome. FFR_CT_ testing in patients with LMCAD is feasible. LM stenosis severity is inversely related to FFR_CT_ value. Patients with LMCAD and FFR_CT_ > 0.80 have favorable clinical outcomes at short-term follow-up. Large-scale studies assessing the clinical utility and safety of deferring invasive catheterization following FFR_CT_ testing in patients with LMCAD are warranted.

**Supplementary Information:**

The online version contains supplementary material available at 10.1007/s10554-021-02371-4.

## Introduction

Left main coronary artery disease (LMCAD) is present in 4–7% of patients undergoing invasive coronary angiography (ICA) [1–3]. Since the presence of LMCAD with stenosis > 50% is associated with unfavorable clinical outcomes, characterization of the left main (LM) anatomy is crucial [4]. Several studies support the use of fractional flow reserve (FFR) to assess the hemodynamic consequences of LMCAD [5–8]. Coronary computed tomography angiography (CTA) is increasingly used as the first line test in patients with suspected coronary artery disease (CAD) [9, 10]. However, as for ICA, CTA findings are often discordant with lesion-specific ischemia as determined by FFR, which currently remains the gold standard for decision-making during ICA [11]. CT-derived FFR (FFR_CT_) has emerged as a test with high diagnostic performance and correlation when compared with measured FFR [11, 12] and as a valuable gatekeeper to the catherization laboratory in patients with stable CAD [13, 14]. Recently, it has been demonstrated that FFR_CT_ is effective in differentiating patients with stenosis who do not require further downstream testing or intervention (FFR_CT_ > 0.80) from higher risk patients in whom further testing and intervention should be considered (FFR_CT_ ≤ 0.80) [15–17]. However, the association between LMCAD and a normal FFR_CT_ result has not previously been explored. Thus, the purpose of this study of patients with stable chest pain was two-fold: 1. to describe the relationship between LMCAD and FFR_CT_ and 2. to evaluate the clinical utility of FFR_CT_ in patients with LMCAD.

## Materials and methods

This single-center, observational all-comer study included patients with LMCAD determined by CTA between November 2015 and December 2017 at Aarhus University Hospital, Denmark. The strategy of CTA as first line testing in symptomatic patients with suspected coronary artery disease (CAD) in this institution has previously been described [14, 15, 18, 19]. In brief, CTA testing is the preferred diagnostic test strategy in patients with non-emergent chest pain and no known CAD such as previous revascularization. FFR_CT_ testing is recommended in patients with one or more lesions of moderate stenosis severity (30 to 70%) before decision-making on downstream management. Direct referral to ICA is generally recommended in patients with high risk anatomy including significant LMCAD, high grade proximal left anterior descending coronary artery (LAD) stenosis, and/or 3-vessel disease in this institution. However, other factors than test results (e.g. clinical presentation, patient preferences, and lesion characteristics) are also considered, when deciding the post-CTA patient management strategy [15]. Therefore, ICA may be deferred in some patients with “high-risk” anatomic features.

### Data sources

Data were retrieved from 3 regional or national registries: 1. the Western Denmark Cardiac Computed Tomography Registry, containing information on the testing indication, patient demographics, CT acquisition characteristics, and CT test results [9], 2. the Danish National Patient Registry providing information on diagnoses, test utilization, and procedures from every hospitalization and outpatient clinical visits [9], and 3. the Civil Registration System, which contains complete data on mortality [9]. The study was approved by the Danish Data Protection Agency (1-16-02-110-17) with a waiver for individual informed consent by the regional ethical committee.

### Coronary CTA

Coronary CTA was performed using dual source scanners (Siemens Definition Flash or Siemens Definition Force, Siemens, Forchheim, Germany) as previously described [14, 15, 18, 19]. In brief, scans were performed according to best CTA acquisition practice guidelines [20]. Oral and/or intravenous beta-blockers or oral ivabradine were administered if necessary, targeting a heart rate < 60 beats/minute. Sublingual spray nitroglycerin 0.8 mg 3 to 5 min before the scan was administered in all patients. An initial non-enhanced high-pitch spiral acquisition scan was performed for assessment of the Agatston score. Coronary CTA acquisition was performed using prospective electrocardiographic triggering. In case of a heart rate of < 65 beats/min a RR scan interval of 65 to 75% was applied and in case of a heart rate of ≥ 65 beats/min the RR acquisition window was widened to 40 to 70%. Vessels with a diameter ≥ 1.8 mm were evaluated for lumen narrowing. Coronary stenosis severity was categorized into four groups: 1–24%, 25–49%, 50–69%, and 70–99%. Stenosis severity ≥ 50% was definded as significant. Stenosis location was defined as proximal or distal as previously described [21]. Patients without a LM (separate ostia), and those with LM or proximal vessel occlusion were not included in this study. Cardiologists with a mean CTA interpretation experience of 7 years performed the readings.

### CTA-derived fractional flow reserve

The science behind FFR_CT_ has previously been described in detail [22]. Standard CTA datasets were transferred for off-site analysis as previously described (HeartFlow, Redwood City, California, US) [11]. A 3D FFR_CT_ model provides computed FFR values in all segments with a lumen diameter > 1.8 mm. A lesion with an FFR_CT_ value ≤ 0.80 was categorized as hemodynamically significant. From November 2015, a 3D interactive FFR_CT_ model was available providing FFR_CT_ values at all points of the coronary tree.

### LMCAD assessment

**Fig. 1:**
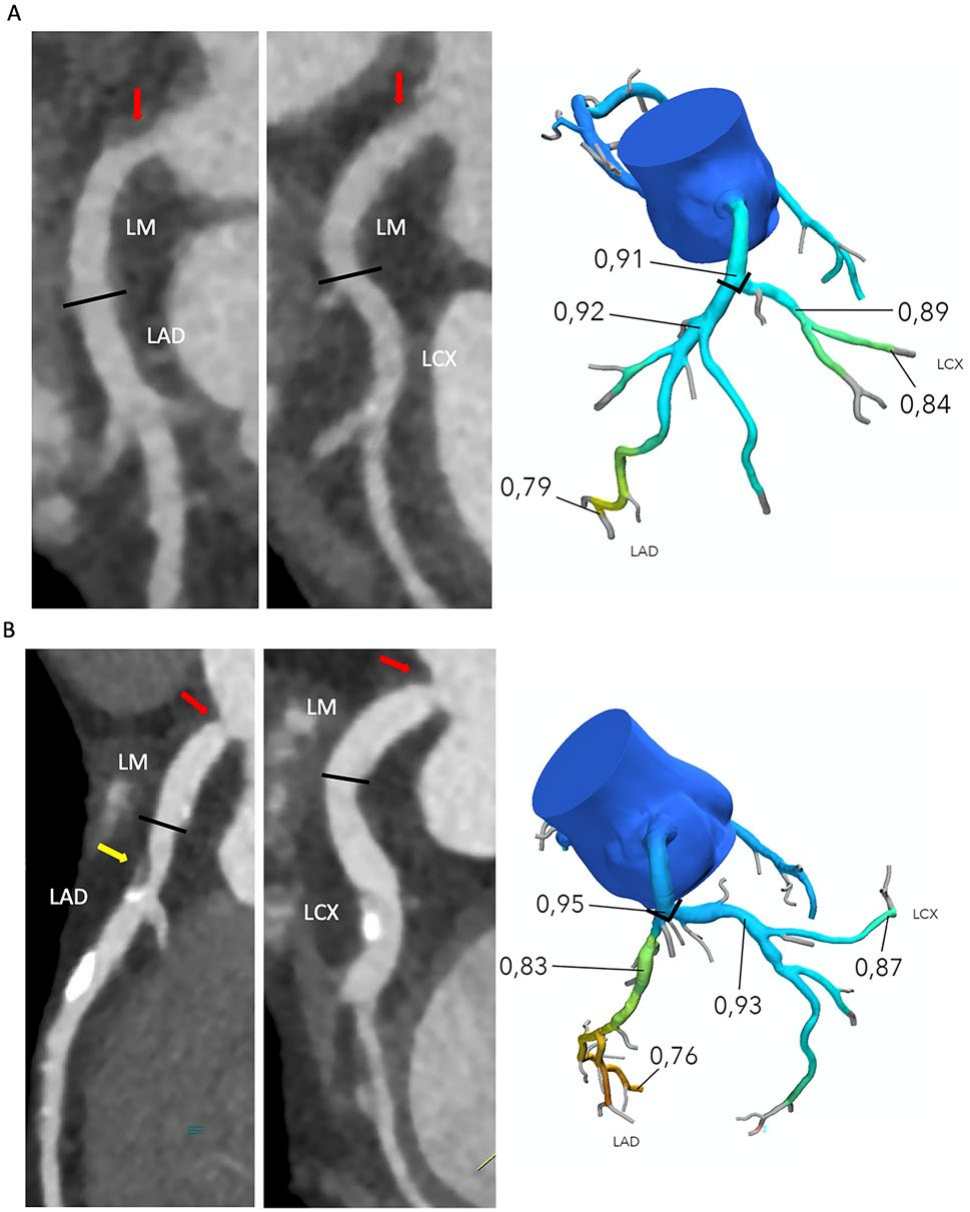
FFR_CT_ reading strategy. Examples of patients with simple (**A**) or complex (**B**) left main coronary artery disease (LMCAD). FFR_CT_ was registered 1. distally in the LM 2. in the proximal left anterior descending (LAD) and left circumflex artery (LCX) segments, and 3. distal segments. The first diagonal branch and first obtuse branch delineated proximal and non-proximal segments. Distal values were assessed in the most distal segments with lumen diameter > 1.8 mm. In patients with simple LMCAD (**A**) FFR_CT_ was registered in all segments 1–3. In patients with complex LMCAD (**B**) downstream FFR_CT_ were registered only in non-stenotic arteries. Thus, FFR_CT_ values in example B were registered only in the distal LM, and proximal and mid LCX segments. Left: Coronary CT angiography curved multiplanar reconstructions. Right: Three-dimensional FFR_CT_ model. The red arrows indicate the location of LMCAD. The yellow arrow denotes a proximal 60% diameter stenosis in the LAD

In patients with simple LMCAD (isolated LM lumen diameter reduction between 1 and 99% with no ≥ 50% stenosis in the left major arteries), absolute FFR_CT_ values were registered, 1. distally in the LM just proximal to the bifurcation when the distal border of the lesion was located ≥ 5 mm from the bifurcation, 2. in the proximal LAD and left circumflex artery (LCx) 1–2 cm distal from the bifurcation, and 3. in the distal LAD and distal LCx segments (Fig. 1). The first diagonal branch, and first obtuse marginal branch were used as delineators between proximal and non-proximal LAD and LCx segments, respectively. Distal FFR_CT_ values were assessed in the most distal LAD and LCx segments (with lumen diameter > 1.8 mm). In patients with complex LMCAD (LMCAD with one or more significant ≥ 50% stenosis in non-LM coronary arteries), downstream FFR_CT_ was registered only in segments without stenosis ≥ 50%. In patients with stenosis ≥ 50% in the proximal part of LAD or LCx, FFR_CT_ was only registered in the non-diseased vessel. An increase of the FFR_CT_ value ≥ 0.03 in mid-proximal relative to more the value in the LM was defined as pressure recovery. Patients with at least one severe lesion with FFR_CT_ < 0.65 in either the proximal LAD or proximal LCx were excluded from the analysis, because FFR < 0.65 in lesions located in proximal segments may influence the reliability of the FFR assessment of the LM [6, 7].

### Clinical endpoint and follow-up

We used a composite endpoint comprising all-cause death, myocardial infarction, and unplanned revascularization. Unplanned revascularization was defined as a procedure performed during an ICA which was not scheduled in the immediate post-CTA FFR_CT_ testing management plan. Follow-up began at the time of the CT scan and continued until the clinical event or end of the study period, June 18, 2018. There was no loss to follow-up during the study period.

### Statistics

Categorical variables were described by counts and percentages. Groups were compared using Fisher´s exact test. Continuous variables were described using mean ± standard deviation (SD) or median (interquartile range, range) as appropriate. Means were compared between the groups using the Student’s t-test with unequal variance and medians were compared using Mann–Whitney U test. Means and medians between more than two groups were compared using the Kruskal–Wallis test. A p value ≤ 0.05 was considered statistically significant. The endpoint analysis was estimated using the Kaplan Meier method. All analysis were performed using SPSS version 25 (SPSS Inc; Chicago, IL, US).

## Results

**Fig. 2:**
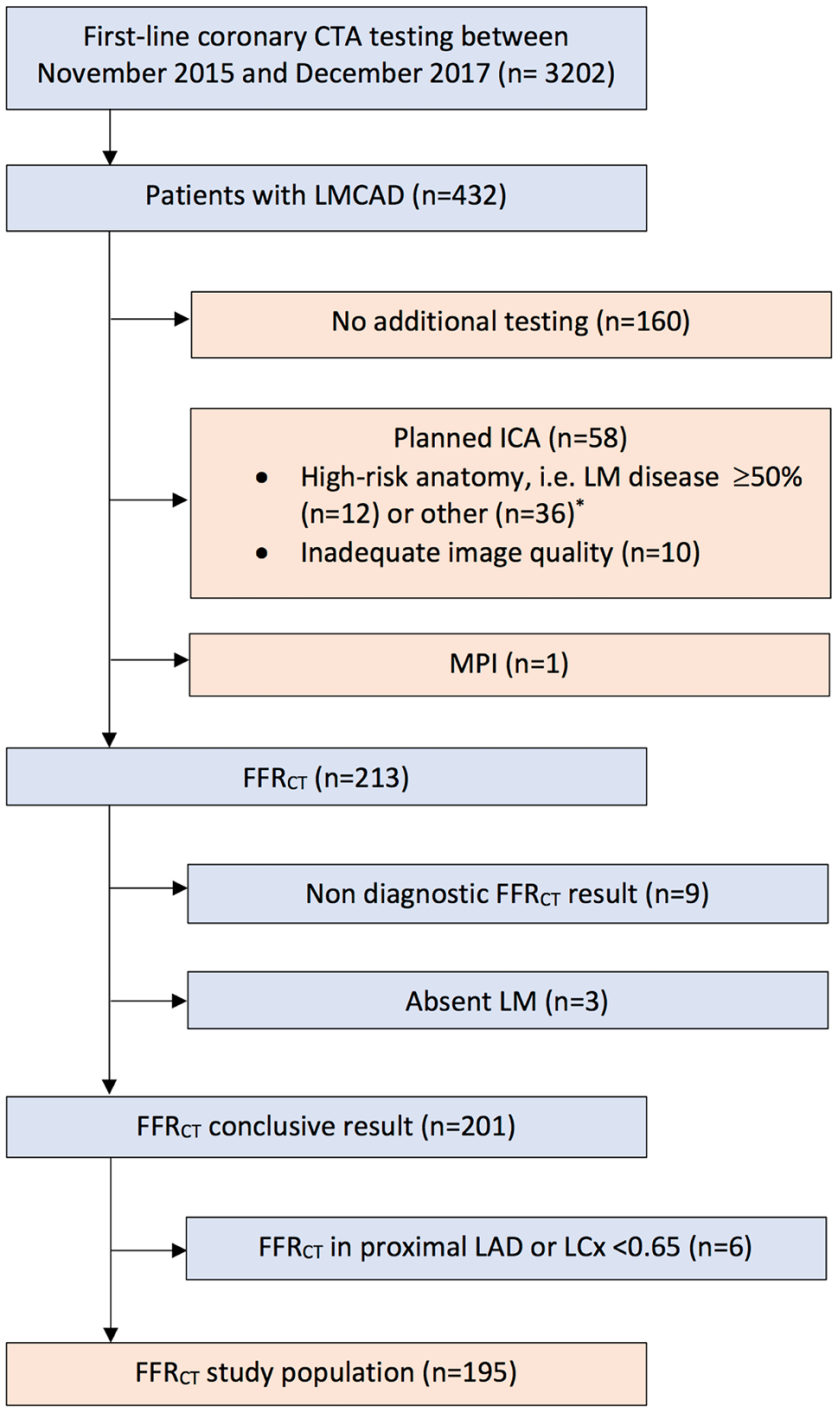
Flow chart of study patients. *CTA* computed tomography angiography, *LMCAD* left main coronary artery disease, *ICA* invasive coronary angiography, *MPI* myocardial perfusion imaging, *FFR*_*CT*_ coronary CTA-derived fractional flow reserve, *LM* left main coronary artery, *prox.* Proximal, *LAD* left anterior descending artery, *LCx* left circumflex artery. *Patients with 3-vessel disease. In patients with LMCAD stenosis ≥ 50%, 9 had 3-vessel disease

During the study period, coronary CTA was performed in 3202 patients. LMCAD was registered in 432 (13%) patients (Fig. 2). Post-coronary CTA direct referral to ICA or myocardial perfusion imaging (MPI) was planned in 59 (14%) patients. Of the remaining patients, FFR_CT_ was prescribed in 213 (49%) patients, while in 160 (37%) patients no additional downstream testing was planned. A conclusive FFR_CT_ result was available in 201 (94%) patients. Coronary CTA image quality was inadequate for FFR_CT_ analysis in 9 (4%) patients, LM was absent in 3 patients, while in 6 patients the FFR_CT_ value of the proximal LAD or LCx was < 0.65 (Fig. 2). Therefore, 195 (45%) patients with available FFR_CT_ results comprised the basis of this report. Baseline patient characteristics are presented in Table 1. Mean (SD) age was 64 (± 10) years, and 62% were men. Patients in the FFR_CT_ group had a higher clinical risk score (Updated Diamond-Forrester, 51% vs. 39%, p < 0.001), and more frequently had typical angina (58% vs. 15%, p < 0.001) than patients in whom FFR_CT_ was not prescribed. Baseline anatomical characteristics of study patients are presented in Table 2. Patients in the FFR_CT_ group had higher median Agatston scores than those in the no FFR_CT_ group (351 vs. 47, p < 0.001), but lower than the group of patients who were referred directly to ICA or myocardial perfusion imaging (351 vs 535, p = 0.009). Coronary CTA acquisition characteristics are presented in Table S1.

**Table 1:** Baseline characteristics

	Total (n = 414)	FFR_CT_ (n = 195)	No further testing (n = 160)	ICA or MPI^a^ (n = 59)	p value^b^
Age, years	64 ± 10	65 ± 9	62 ± 10	65 ± 10	0.03
Male	256 (62)	122 (63)	88 (55)	46 (78)	0.16
Diabetes mellitus	44 (11)	17 (9)	12 (8)	15 (25)	0.71
Hypertension	176 (43)	84 (43)	59 (37)	33 (56)	0.07
Hyperlipidemia	167 (40)	74 (38)	70 (44)	23 (39)	0.25
Current smoker	93 (23)	48 (25)	31 (19)	14 (24)	0.59
Family history of CAD	167 (40)	75 (39)	62 (39)	30 (51)	1.00
Updated Diamond–Forrester risk score, %	47 ± 21	51 ± 21	39 ± 18	59 ± 19	< 0.001
Angina					< 0.001
Typical angina	99 (24)	58 (30)	15 (9)	26 (44)	
Atypical angina	266 (64)	126 (65)	113 (71)	27 (46)	
Serum creatinine, μmol/l	79 ± 21	79 ± 20	77 ± 18	86 ± 30	0.50

Values are mean ± SD or numbers (%)

*CAD* coronary artery disease, *FFR*_*CT*_ coronary CTA-derived fractional flow reserve, *ICA* invasive coronary angiography, *MPI* myocardial perfusion imaging

^a^Patients referred directly to ICA (n = 58) or MPI (n = 1) without FFR_CT_

^b^Comparison between the groups of FFR_CT_ and No FFR_CT_

**Table 2:** Anatomical characteristics

	Total (n = 414)	FFR_CT_ (n = 195)	No further testing (n = 160)	ICA or MPI^a^ (n = 59)	p value^b^
Agatston score	209 (38–539, 0–4904)	351 (130–737, 0–4904)	47 (6–207, 0–1394)	535 (221–1114, 5–2940)	< 0.001
LM stenosis 1–24%	274 (66)	104 (53)	141 (88)	29 (49)	< 0.001
LM stenosis 25–49%	108 (26)	72 (37)	18 (11)	18 (31)	
LM stenosis 50–69%	28 (7)	17 (9)	1 (1)	10 (17)	
LM stenosis 70–99%	4 (1)	2 (1)	0	2 (3)	
LM stenosis 1–49%					< 0.001
Simple LMCAD	173 (42)	13 (7)	154 (96)	6 (10)	
Complex LMCAD	209 (51)	163 (84)	5 (3)	41 (70)	
LM stenosis 50–99%					
Simple LMCAD	9 (2)	8 (4)	0	1 (2)	
Complex LMCAD	23 (6)	11 (6)	1 (1)	11 (19)	

Values are numbers (%) or median (interquartile range, range)

*FFR*_*CT*_ Coronary CTA-derived fractional flow reserve, *ICA* invasive coronary angiography, *MPI* myocardial perfusion imaging, *LM* left main, *LMCAD* left main coronary artery disease, *Simple LMCAD* isolated left main disease, *Complex LMCAD* left main disease with one or more significant stenosis in non LM coronary arteries

^a^Patients referred directly to ICA (n = 58) or MPI (n = 1) without FFR_CT_

^b^Comparison between the groups of FFR_CT_ and No FFR_CT_

### Relationship between left main anatomy and downstream physiology

In patients undergoing FFR_CT_ testing, maximum LM stenosis ranged between 1–24%, 25–49%, 50–69% and 70–99% in 53%, 37%, 9% and 1%, respectively. FFR_CT_ values in the distal LM, proximal LAD, proximal LCx, distal LAD and distal LCx decreased with increasing LM stenosis severity (Table 3).

**Table 3:** FFR_CT_ values in the distal LM, proximal LAD and LCx and distal LAD and LCx according to LM stenosis severity

LM stenosis severity	FFR_CT_ study population (n = 195)				p value^a^
	1–24% (n = 104)	25–49% (n = 72)	50–69% (n = 17)	70–99% (n = 2)	
FFR_CT_ distal LM	0.97 (0.96–0.98, 0.89–0.99) (n = 104, FFR_CT_ ≤ 0.80 n = 0)	0.96 (0.93–0.97, 0.73–1.00) (n = 72, FFR_CT_ ≤ 0.80 n = 2)	0.91 (0.85–0.95, 0.70–0.98) (n = 17, FFR_CT_ ≤ 0.80 n = 1)	0.87 (n = 2, FFR_CT_ ≤ 0.80 n = 0)	< 0.001
FFR_CT_ proximal LAD	0.95 (0.93–0.96, 0.88–0.99) (n = 68, FFR_CT_ ≤ 0.80 n = 0)	0.93 (0.89–0.94, 0.72–0.98) (n = 43, FFR_CT_ ≤ 0.80 n = 3)	0.90 (0.79–0.94, 0.67–0.95) (n = 9, FFR_CT_ ≤ 0.80 n = 3)	0.84 (n = 2, FFR_CT_ ≤ 0.80 n = 0)	< 0.001
FFR_CT_ distal LAD	0.82 (0.77–0.86, 0.50–0.95) (n = 68, FFR_CT_ ≤ 0.80 n = 32)	0.81 (0.72–0.85, 0.50–0.92) (n = 39, FFR_CT_ ≤ 0.80 n = 19)	0.78 (0.66–0.89, 0.62–0.91) (n = 9, FFR_CT_ ≤ 0.80 n = 5)	0.62 (n = 2, FFR_CT_ ≤ 0.80 n = 2)	0.23
FFR_CT_ proximal LCx	0.96 (0.94–0.97, 0.84–0.99) (n = 87, FFR_CT_ ≤ 0.80 n = 0)	0.93 (0.90–0.95, 0.74–0.98) (n = 59, FFR_CT_ ≤ 0.80 n = 4)	0.91 (0.84–0.95, 0.67–0.97) (n = 14, FFR_CT_ ≤ 0.80 n = 1)	0.82 (n = 1, FFR_CT_ ≤ 0.80 n = 0)	< 0.001
FFR_CT_ distal LCx	0.90 (0.84–0.92, 0.51–0.95) (n = 86, FFR_CT_ ≤ 0.80 n = 11)	0.85 (0.80–0.91, 0.60–0.96) (n = 60, FFR_CT_ ≤ 0.80 n = 17)	0.85 (0.77–0.93, 0.61–0.95) (n = 14, FFR_CT_ ≤ 0.80 n = 6)	0.79 (n = 1, FFR_CT_ ≤ 0.80 n = 1)	0.03

Values are median (interquartile range, range). Numbers in columns do not sum up to the total number of patients in each column header because in patients with complex LMCAD (LMCAD with one or more significant ≥ 50% stenosis in non-LM coronary arteries), downstream FFR_CT_ values were registered only in segments without stenosis ≥ 50%. In patients with stenosis ≥ 50% in the proximal part of LAD or LCx, FFR_CT_ was only registered in the non-diseased vessel

*FFR*_*CT*_ Coronary CTA-derived fractional flow reserve, *LM* left main coronary artery, *LAD* left anterior descending artery, *LCx* left circumflex artery

^a^Comparison between all groups

In patients with simple LMCAD (n = 21) and complex LMCAD (n = 174) FFR_CT_ values were significantly lower in the group with LM stenosis ≥ 50% versus those without stenosis (Table S2). Yet FFR_CT_ in the LM was > 0.80 in 95% (18/19) of the patients with maximum LM stenosis ≥ 50%, including 7 (39%) with simple LMCAD and 11 (61%) with complex LMCAD, respectively. The proportion of patients with maximum LM stenosis ≥ 50%, and FFR_CT_ > 0.80 decreased to 82% (9/11) when FFR_CT_ was assessed in non-diseased proximal LAD and LCx segments. The number of patients with a significant FFR_CT_ value was highest in the distal segments (Table S3).

**Fig. 3:**
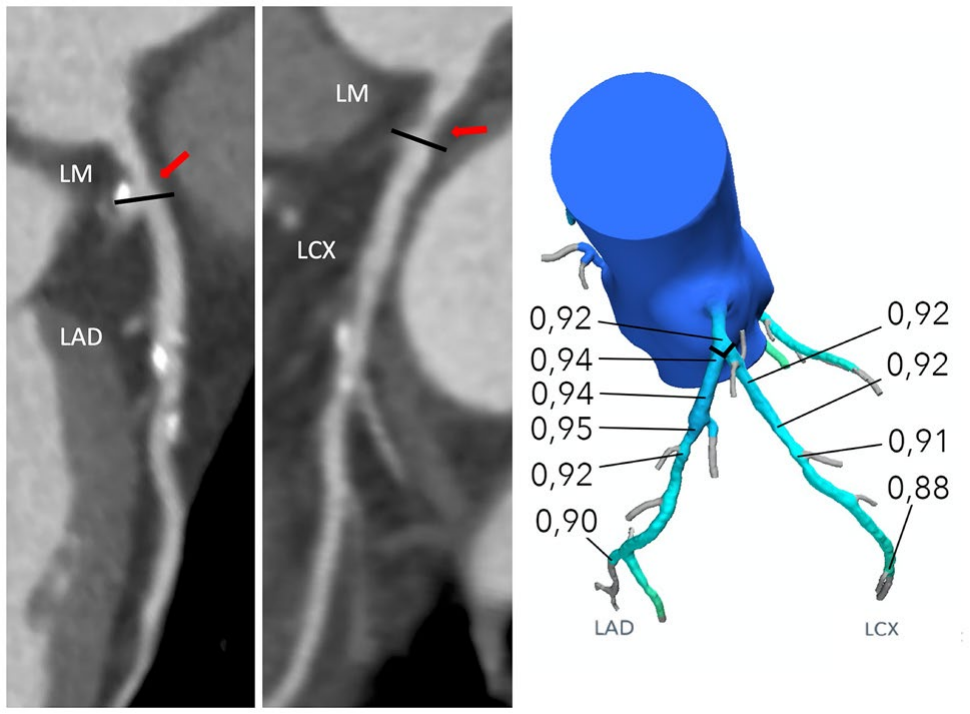
Pressure recovery phenomenon. Typically, pressure will reach a minimum in the throat of stenosis with slight pressure recovery 0.5–1 cm distal to the stenosis because of the increase in the cross-sectional area of the vessel and then decrease further downstream the vessel due to the continuous decrease in the cross-sectional area of the vessel and possibly the presence of flow limiting artery disease in more distal segments. However, FFR_CT_ values may transiently rise also in segments located more distal to stenosis. In this case, the step-up in FFR_CT_ from 0.92 distally in the left main (LM) to 0.95 in the mid left anterior descending artery (LAD) is caused by the presence of post-stenotic vessel dilatation resulting in reduced flow velocity and pressure recovery. We defined significant pressure recovery as an increase in FFR_CT_ ≥ 0.03 when moving from the lesion-specific FFR_CT_ "reading point" (typically 1–2 cm distal to the lower border of the stenosis) to more distal located segments. Coronary CT angiography curved multiplanar reconstructions. Right: Three-dimensional FFR_CT_ model. Red arrow indicates the location of LMCAD. *LCX* left circumflex artery

In 3 (2%) patients pressure recovery was identified; downstream FFR_CT_ values > 0.80 in all. One such case is presented in Fig. 3.

### Clinical outcomes

The risk of the composite endpoint during follow-up was 5% (Table 4). There was a numerically but not statistically significant difference in the risk of the composite endpoint when comparing the FFR_CT_ and no FFR_CT_ groups (5% versus 1%, p = 0.15) as shown in Table S4.

**Table 4:** Clinical composite endpoint according to anatomical and physiological characteristics

	FFR_CT_ study population (n = 195)	Simple LMCAD, LM stenosis 1–49%		Simple LMCAD, LM stenosis 50–99%		Complex LMCAD, LM stenosis 1–49%		Complex LMCAD, LM stenosis 50–99%		p value^a^
		FFR_CT_ LM ≤ 0.80 (n = 0)	FFR_CT_ LM > 0.80 (n = 13)	FFR_CT_ LM ≤ 0.80 (n = 1)	FFR_CT_ LM > 0.80 (n = 7)	FFR_CT_ LM ≤ 0.80 (n = 2)	FFR_CT_ LM > 0.80 (n = 161)	FFR_CT_ LM ≤ 0.80 (n = 0)	FFR_CT_ LM > 0.80 (n = 11)	
Composite endpoint	9 (5)	0	0	0	0	0	8 (5)	0	1 (9)	0.90
All-cause death	3 (2)	0	0	0	0	0	2 (1)	0	1 (9)	0.78
Myocardial infarction	3 (2)	0	0	0	0	0	3 (2)	0	0	0.99
Unplanned revascularization	3 (2)	0	0	0	0	0	3 (2)	0	0	0.99
Total number of revascularizations	52 (27)	0	0	1 (100)	2 (29)	1 (50)	41 (26)	0	7 (64)	0.005
ICA										0.03
Planned ICA	79 (41)	0	1 (8)	1 (100)	3 (43)	1 (50)	65 (40)	0	8 (73)	
Unplanned ICA	6 (3)	0	1 (8)	0	2 (29)	0	3 (2)	0	0	

Values are n (%)

*LMCAD* left main coronary artery disease, *LM* left main, *FFR*_*CT*_ coronary CTA-derived fractional flow reserve, *ICA* invasive coronary angiograph

^a^Comparison across all groups

The risk of the composite endpoint, the number of ICA and of revascularization procedures in patients with simple or complex LMCAD based on the LM FFR_CT_ values are presented in Table 4. There were no events in patients with simple LMCAD, of whom 7 of 8 with LM stenosis ≥ 50% had FFR_CT_ > 0.80. The number of ICAs and revascularizations according to the anatomical findings and FFR_CT_ results in patients with simple LMCAD are shown in Fig. S1. Patients with simple LMCAD with stenosis ≥ 50% having ICAs or revascularizations performed were more likely to have numerically lower FFR_CT_ values than those without ICA or revascularization.

In a subanalysis including the total FFR_CT_ cohort and distal FFR_CT_ values the risk of composite endpoint was 6% (7/126) vs. 3% (2/69) in patients with distal FFR_CT_ value ≤ 0.80 and FFR_CT_ value > 0.80, respectively (p = 0.09) (Table S5 and Fig. S2).

## Discussion

In this study of consecutive symptomatic patients undergoing first line coronary CTA, LM stenosis severity was inversely related to FFR_CT_ values irrespective of the reading point i.e. in the distal LM, or the proximal or distal LAD or LCx segments. In patients with simple LMCAD and stenosis ≥ 50%, more than 80% had FFR_CT_ > 0.80 in non-diseased proximal and distal LAD and/or LCx segments. FFR_CT_ > 0.80 in patients with LMCAD was associated with favorable clinical outcomes.

Left main artery stenosis is associated with unfavorable outcomes. Therefore, societal guidelines emphasize the importance of revascularization of LM stenosis [23]. Although, FFR represents the gold standard for decision-making in the catherization laboratory, guidelines recommend that in the event of significant LM disease that treatment decision-making is guided by intravascular ultrasound (IVUS) or optical coherence tomography (OCT) (IIa recommendation level) [23].

Patients with significant LMCAD were excluded from the FAME trials [24, 25], however preliminary data indicate that long-term outcome is more favorable in patients undergoing FFR than pure angiographically guided LM revascularization [8]. Assessing LM disease based on angiography or physiology is challenging due to the short length, catheter damping [26] and overlap of downstream vessels [27]. Moreover, downstream disease in the proximal LAD or LCx may influence the FFR values over LM stenosis potentially leading to false negative results [7, 28]. Accordingly, it has been demonstrated that FFR < 0.65 in lesions located in the proximal segments may influence the reliability of FFR assessment of LMCAD [6, 7].

Coronary CTA is increasingly used as the first line test in patients suspected of stable CAD, a strategy which is supported by guidelines [10]. Recently, the evidence for FFR_CT_ has expanded beyond diagnostic validation [11, 12] by facilitating less referrals to ICA and less findings of non-obstructive disease in patients with moderate CAD [13–15]. Moreover, it has recently been demonstrated that patients with intermediate stenosis and FFR_CT_ > 0.80 have favorable clinical outcomes without the need of further testing [15–17]. However, no previous study has investigated the potential clinical utility of FFR_CT_ in patients with LMCAD. In this institution, patients with significant LM stenosis by coronary CTA are categorized as high risk and therefore per institutional practice it is recommended that such patients are referred directly to ICA, while in patients with non-obstructive LMCAD FFR_CT_ may be used for non-invasive hemodynamically adjudication. However, other circumstances than LM stenosis severity may have influenced downstream clinical decision-making in these patients, such as symptoms, other lesion anatomical characteristics, and patient preferences. Therefore, in this study FFR_CT_ was used as an adjunctive test before decision-making on downstream management even in some patients with simple LM stenosis. In some of these patients, ICA was deferred based on a normal FFR_CT_ result.

While FFR interrogation for assessment of LMCAD is performed in the proximal LAD and LCx segments [5] in this study, we also registered, FFR_CT_ values in the distal LM. In accordance with previous findings we found that FFR_CT_ values were inversely associated with LM stenosis severity [15]. Moreover, FFR_CT_ values were lower in distal than in proximal segments reflecting the fact that FFR_CT_ is the sum of multiple downstream resistances from discrete lesions or diffuse disease. One striking finding was the low proportion of FFR_CT_ positivity in significant LM stenosis, even among those with simple LMCAD with stenosis ≥ 50%. In patients with simple LMCAD and stenosis ≥ 50% only 13% and 13% demonstrated FFR_CT_ ≤ 0.80 when assessed distally in the LM or in proximal LAD and LCx segments. In comparison, in another real-world report from this institution, the proportion of stable patients with stenosis ≥ 50% in proximal segments and FFR_CT_ ≤ 0.80 was 48% [15]. In a previous study assessing LMCAD physiology, it was demonstrated that significant stenosis or lesions in the downstream vessels may result in overestimation of FFR values [28]. However, since the low proportion of FFR_CT_ positivity was present even in the event of minimal or absent downstream disease other mechanisms most likely play a role. The short length of the LM may potentially influence the atherosclerotic plaque formation and reliability of diameter stenosis assessment [29]. Rheological factors in very proximal LM stenosis may also play a role. At the entry of the left coronary system the blood flow is turbulent and pressure losses reduced compared to segments with laminar flow [29]. These findings need further delineation in future studies.

In contrast to FFR, which measures pressure at the location of the pressure wire, FFR_CT_ values are available everywhere in the coronary tree. Thus, unlike invasive FFR, FFR_CT_ may potentially be assessed in the LM stem. However, due to the short length of the LM it may be difficult in the majority of patients to obtain the value 10–20 mm distal to the lower border of stenosis, which is the location recommended for management actions [30]. A shorter distance between the lesion and the FFR_CT_ reading point may potentially lead to more cases with pressure recovery which typically occurs just distal to a stenosis due to the increase in the cross-sectional area and corresponding loss in flow velocity (Bernoulli’s principle) and then decreases again due to the continuous decrease in vessel area and/or downstream disease. The phenomenon was infrequently seen in this dataset, and thus could not explain the high number of FFR_CT_ > 0.80 in patients with LM stenosis ≥ 50%.

Importantly, outcomes were favorable in patients with simple LMCAD and FFR_CT_ ≥ 0.80. However, these findings are exploratory only and thus need confirmation in future studies. Overall, the proportion of patients with an adverse cardiac event during short-term follow-up was low. Notably, all adverse events occurred in patients with complex LMCAD. More studies are needed to assess the clinical utility of FFR_CT_ in patients with LMCAD including exploration of the safety of deferring ICA in those with FFR_CT_ > 0.80 as well as assessing the value of FFR_CT_ ≤ 0.80 for decision-making on ICA and revascularization.

LM disease has traditionally required invasive angiography with or without FFR for determination of revascularization. CTA has up till now been deemed unable to adequately assess patients with LM disease. Potentially, the inherent risk of periinterventional complications in LMCAD patients may be reduced if safety of CTA-FFR_CT_ assessment for deferral of ICA in this setting can be confirmed in larger studies. This is a first description of the feasibility and clinical outcomes of FFR_CT_ use in patients with LM disease. The present data are in accordance with recent data demonstrating the promise of extension of CTA use in more complex lesion subsets, and in those which had been previously deemed inappropriate or impossible for CTA testing [31, 32].

### Study limitations

This is a single-center study with inherent limitations such as selection bias and possibly lack of generalizability of results. The number of patients with LM stenosis ≥ 50% was limited. However, the present study included an all-comer consecutive cohort of symptomatic patients, and thus is representative of patients encountered in clinical practice. In this study individual CT cardiologists prescribed FFR_CT_ or ICA according to a varying degree of integrating test preferences and thresholds. We have no further information about reasons for sending some patients directly to ICA and not to FFR_CT_ testing and vice versa. The proportion of patients with significant LMCAD or adverse clinical outcomes was low. Information of angina would have been valuable. Studies with more patients and longer follow-up are needed to confirm the present findings.

## Conclusions

FFR_CT_ testing in patients with LMCAD is feasible. LM stenosis severity is inversely related to downstream FFR_CT_ values. Patients with LMCAD and FFR_CT_ > 0.80 have favorable clinical outcomes at short-term follow-up. More studies assessing the clinical utility and safety of FFR_CT_ testing in patients with LMCAD are warranted.

## Supplementary Information

Below is the link to the electronic supplementary material.

Supplementary file1 (PDF 330 kb)

## Data Availability

The study data supporting the manuscript are available from the corresponding author upon approval of a reasonable request.
