# Supplementary material for: Coronary CT angiography derived FFR in patients with left main disease
Source: Int J Cardiovasc Imaging. 2021 Aug 12;37(11):3299–308. doi: 10.1007/s10554-021-02371-4 (PMC8557153; doi:10.1007/s10554-021-02371-4)
Supplement: Supplementary file 1 — Supplementary file1 (PDF 330 kb) [file 10554_2021_2371_MOESM1_ESM.pdf]

Supplementary data:

Table S1 Coronary CTA Acquisition Characteristics According to the Downstream Testing Strategy

	Total<br>(n=414)	FFR_{CT}<br>(n=195)	No FFR_{CT}<br>(n=160)	ICA or MPI[a]<br>(n=59)	p Value[b]
Betablockers or Ivabradine	335 (81)	152 (78)	136 (85)	47 (80)	0.10
Mean heart rate, bpm	59 ± 12	57 ± 8	59 ± 14	63 ± 15	0.90
Sinus rhythm at CTA	399 (96)	191 (98)	150 (94)	58 (98)	0.06
Contrast, mL	66 ± 13	68 ± 16	64 ± 8	64 ± 10	0.01
Mean diagnostic radiation dose exposure, mSv	3.7 ± 2.3	3.9 ± 2.5	3.5 ± 2.1	3.6 ± 1.8	0.35

Values are mean ± SD or numbers (%). [a]Patients referred directly to ICA (n=58) or MPI (n=1) without FFR_{CT}. [b]Comparison between the groups of FFR_{CT} and no FFR_{CT}. CTA= computed tomography angiography; FFR_{CT} = CT derived fractional flow reserve; ICA = invasive coronary angiography; MPI = myocardial perfusion imaging.

Table S2 FFR_{CT} Values in Patients with Simple or Complex LMCAD

	FFR_{CT} distal LM	FFR_{CT} proximal LAD	FFR_{CT} distal LAD	p Value[a]	FFR_{CT} proximal LCx	FFR_{CT} distal LCx	p Value[b]
Simple LMCAD with LM stenosis 1-49%	0.97 (0.95-0.98, 0.87-0.99) n=13	0.94 (0.93-0.96, 0.83-0.99) n=13	0.84 (0.80-0.90, 0.50-0.91) n=13	< 0.001	0.97 (0.94-0.98, 0.89-0.99) n=13	0.91 (0.89-0.93, 0.77-0.94) n=13	< 0.001
Simple LMCAD with LM stenosis 50-99%	0.91 (0.82-0.95, 0.70-0.98) n=8	0.89 (0.80-0.94, 0.67-0.95) n=8	0.78 (0.69-0.89, 0.62-0.91) n=8	< 0.001	0.92 (0.82-0.94, 0.67-0.97) n=8	0.87 (0.78-0.93, 0.61-0.94) n=8	0.003
Complex LMCAD with LM stenosis 1-49%	0.97 (0.95-0.98, 0.73-1.00) n=163	0.94 (0.91-0.96, 0.72-0.98) n=98	0.81 (0.75-0.85, 0.50-0.95) n=94	< 0.001	0.95 (0.92-0.97, 0.74-0.99) n=133	0.87 (0.81-0.92, 0.51-0.96) n=133	< 0.001
Complex LMCAD with LM stenosis 50-99%	0.91 (0.85-0.95, 0.82-0.98) n=11	0.86 n=3	0.63 n=3	0.10	0.88 (0.85-0.96, 0.81-0.96) n=7	0.79 (0.77-0.94, 0.77-0.95) n=7	0.005

Values are median (interquartile range, range). Numbers in columns do not sum up to the total number of patients in each column header because in patients with complex LMCAD (LMCAD with one or more significant ≥50% stenosis in non-LM coronary arteries), downstream FFR_{CT} values were registered only in segments without stenosis ≥50%. In patients with stenosis ≥50% in the proximal part of LAD or LCx, FFR_{CT} was only registered in the non-diseased vessel. [a]Comparison between the groups of FFR_{CT} distal LM, FFR_{CT} proximal LAD and FFR_{CT} distal LAD. [b]Comparison between the groups of FFR_{CT} distal LM, FFR_{CT} proximal LCx and FFR_{CT} distal LCx. LMCAD = Left main coronary artery disease. FFR_{CT} = CT derived fractional flow reserve. LM =left main coronary artery; LAD = left anterior descending artery; LCx = left circumflex artery.

Table S3 Patients with a Significant FFR_{CT} Value According to LM Stenosis Severity and The FFR_{CT} reading point

	Stenosis severity			
	**1-24% (n=104)**	**25-49% (n=72)**	**50-69% (n=17)**	**70-99% (n=2)**
FFR_{CT} distal LM ≤0.80	0	2 (3)	1 (6)	0
FFR_{CT} proximal LAD ≤0.80	0	3 (4)	3 (18)	0
FFR_{CT} distal LAD ≤0.80	32 (31)	19 (26)	5 (30)	2 (100)
FFR_{CT} proximal LCx ≤0.80	0	4 (6)	1 (6)	0
FFR_{CT} distal LCx ≤0.80	11 (11)	17 (24)	6 (35)	1 (50)

Values are numbers (%). Numbers in columns do not sum up to the total number of patients in each column header because in patients with complex LMCAD (LMCAD with one or more significant ≥50% stenosis in non-LM coronary arteries), downstream FFR_{CT} values were registered only in segments without stenosis ≥50%. In patients with stenosis ≥50% in the proximal part of LAD or LCx, FFR_{CT} was only registered in the non-diseased vessel. FFR_{CT} = CT derived fractional flow reserve; LM =left main coronary artery; prox. = proximal; LAD = left anterior descending artery; LCx = left circumflex artery.

Table S4 Clinical Endpoints in the FFR_{CT} Group versus the No FFR_{CT} Group

	**Total** (n=414)	**FFR_{CT}** (n=195)	**No FFR_{CT}** (n=160)	**ICA or MPI**[a] (n=59)	**p Value**[b]
Composite endpoint	17 (4)	9 (5)	1 (1)	7 (12)	0.15
All-cause death	6 (1)	3 (2)	1 (1)	2 (3)	0.71
Myocardial infarction	7 (2)	3 (2)	0	4 (7)	--
Unplanned revascularization	4 (1)	3 (2)	0	1 (2)	--
Total number of revascularizations	96 (23)	52 (27)	0	44 (75)	< 0.001
ICA					< 0.001
Planned ICA	137 (33)	79 (41)	0	58 (98)	
Unplanned ICA	9 (2)	6 (3)	2 (1)	1 (2)	

Values are numbers (%). [a]Patients referred directly to ICA (n=55) or MPI (n=4) without FFR_{CT}. [b]Comparison between the FFR_{CT} and No FFR_{CT} group. ICA = invasive coronary angiography. FFR_{CT} = CT derived fractional flow reserve. MPI = myocardial perfusion imaging.

Table S5 Clinical Adverse Events According to FFR_{CT} Results in the Distal Coronary Segments

	**distal FFR_{CT} values ≤0.80** (n=126)	**distal FFR_{CT} values >0.80** (n=69)	**p Value**[a]
Composite endpoint	7 (6)	2 (3)	0.09
All-cause death	2 (2)	1 (1)	0.71
Myocardial infarction	3 (2)	0	0.55
Unplanned revascularization	2 (2)	1 (1)	1.00

Values are n (%) [a] Comparison between the groups of distal FFR_{CT} values ≤0.80 and >0.80. FFR_{CT} = Coronary CTA-derived fractional flow reserve; ICA = invasive coronary angiography.

Fig. S1 FFR_{CT} Values in Patients with LM Disease According to the Downstream Management Strategy (ICA and Revascularization)

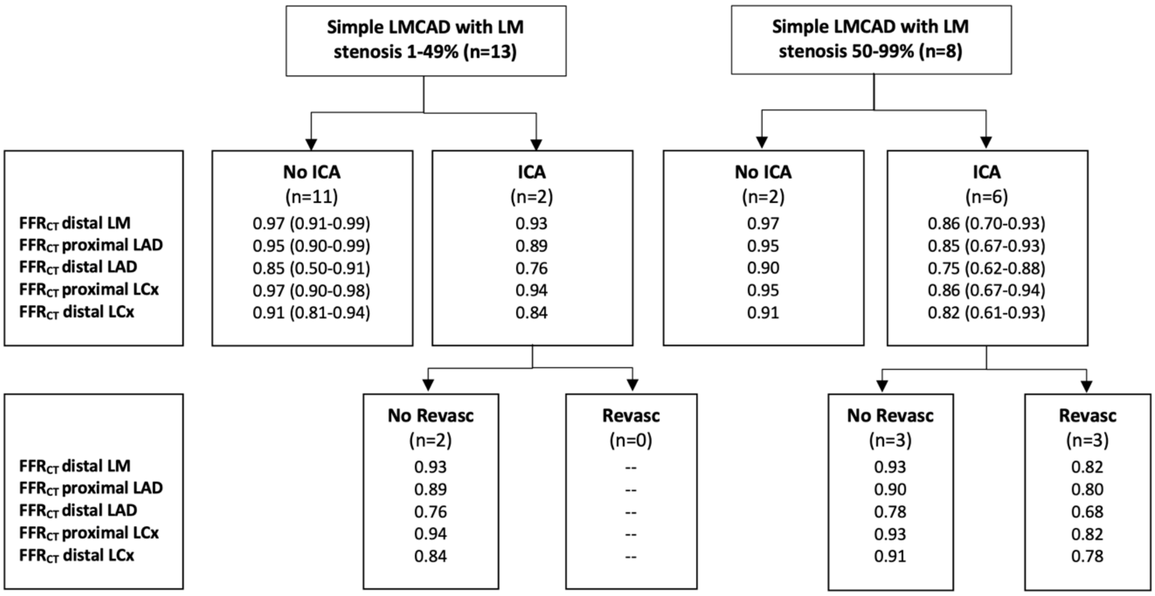

Values are median (range). No ICA = no invasive coronary angiography, ICA = invasive coronary angiography, No Revasc = No revascularization, Revasc = revascularization.

Fig. S2 Clinical Adverse Events as a Function of the FFR_{CT} Values in the Distal Coronary Segments

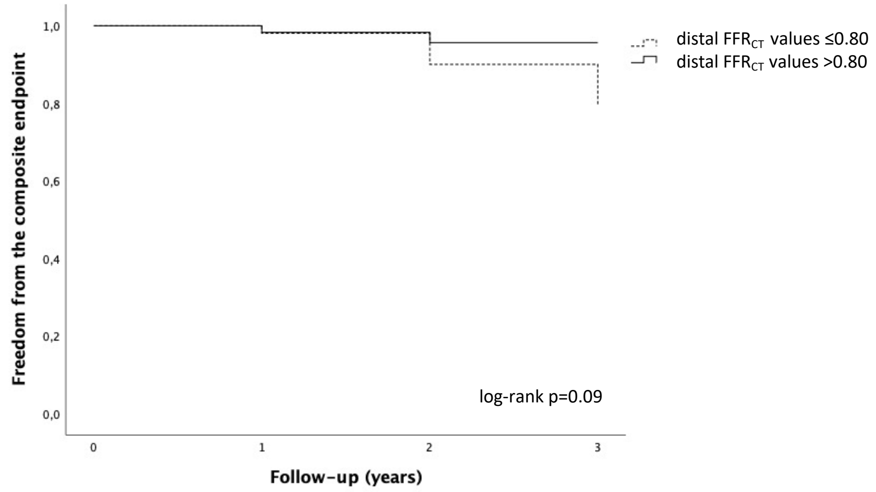

Number at risk

distal FFR_{CT} values ≤0.80	126	78	16
distal FFR_{CT} values >0.80	69	49	24

FFR_{CT} = CT derived fractional flow reserve. Composite Endpoint = all-cause death, myocardial infarction, unplanned revascularization. Distal FFR_{CT} values = distal LAD and/or LCx values.
